# Supplementary material for: Interplay of two transcription factors for recruitment of the chromatin remodeling complex modulates fungal nitrosative stress response
Source: Nat Commun. 2021 May 6;12:2576. doi: 10.1038/s41467-021-22831-8 (PMC8102577; doi:10.1038/s41467-021-22831-8)
Supplement: Supplementary file 1 — Supplementary Information [file 41467_2021_22831_MOESM1_ESM.pdf]

# Interplay of two transcription factors for recruitment of the chromatin remodeling complex modulates fungal nitrosative stress response

Yunqing Jian[1], Zunyong Liu[2], Haixia Wang[1], Yun Chen[1,2], Yanni Yin[1,2], Youfu Zhao[3], Zhonghua Ma[1,2,*]

## Supplementary Information

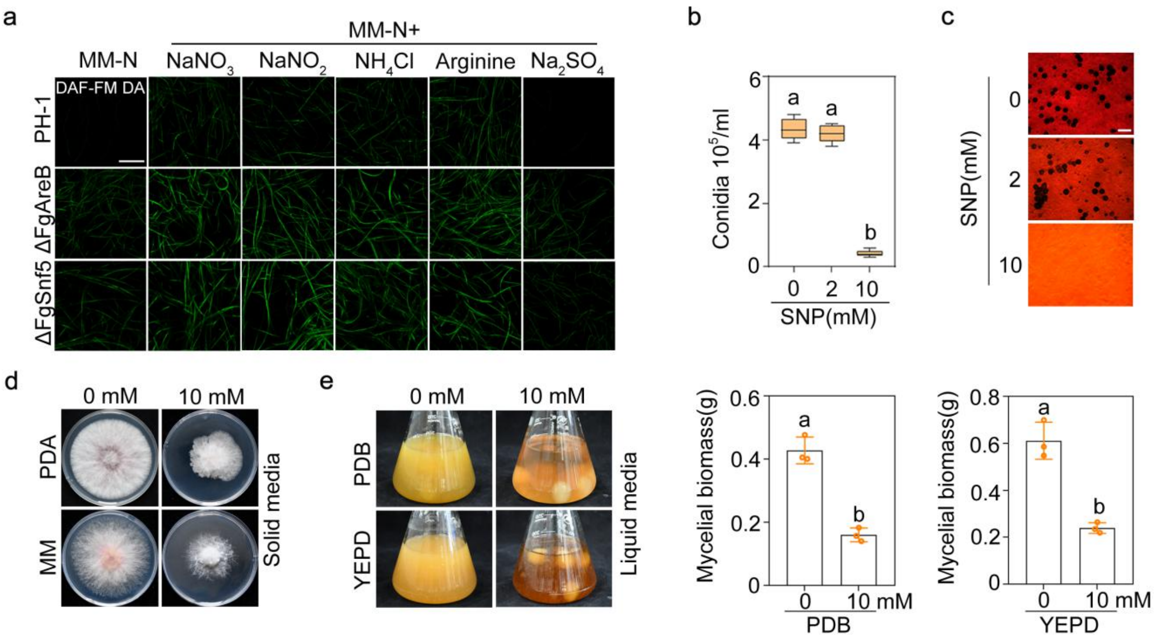


**Supplementary Fig. 1. Nitrosative stress inhibits *F. graminearum* growth and development.**

**(a)** Nitrogen induces NO production in *F. graminearum* hyphae. Hyphae of PH-1 that were cultured in YEPD for 16 hr and subseqently incubated for 4 hr in MM-N or with 10 mM of each indicated chemical were stained with DAF-FM DA. Fluorescence was detected by a confocal microscopy. Bars: 50 μm. The experiment was repeated three times independently with similar results.

**(b)** SNP treatment inhibited *Fg* conidiation. Conidia were quantified using a hemacytometer after incubation of PH-1 strain in CMC containing 0, 2, or 10 mM SNP for four days. Data are shown as box plots with the interquartile range as the upper and lower confines of the box, and the median as a solid line within the box. Different letters indicate statistically significant differences according to the one-way ANOVA test ($p$ <0.05).

**(c)** SNP treatment led to sexual sterility. PH-1 strain was grown on carrot agar with or without SNP for induction of perithecial formation after 20 days. Bars: 500 μm. The

experiment was repeated twice independently with similar results.

**(d)** SNP inhibited the mycelial growth of *F. graminearum* on solid media. A five-mm mycelial plug of PH-1 was inoculated on PDA or MM plate supplemented with or without 10 mM SNP and incubated at 25 °C for three days.

**(e)** SNP inhibited the mycelial growth of *F. graminearum* in liquid media. Six five-mm mycelial plugs of PH-1 were inoculated on PDB or YEPD medium supplemented with or without 10 mM SNP and incubated at 25 °C for 36 hours with 180 rpm (left panel). Dried mycelia (biomasses) were measured for cultures in liquid PDB (middle panel) or YEPD (right panel). Data presented are the mean ± standard errors from three biological replicates (n=3). Different letters represent statistically significant differences according to the two-tailed Student's *t*-test ($p < 0.05$).

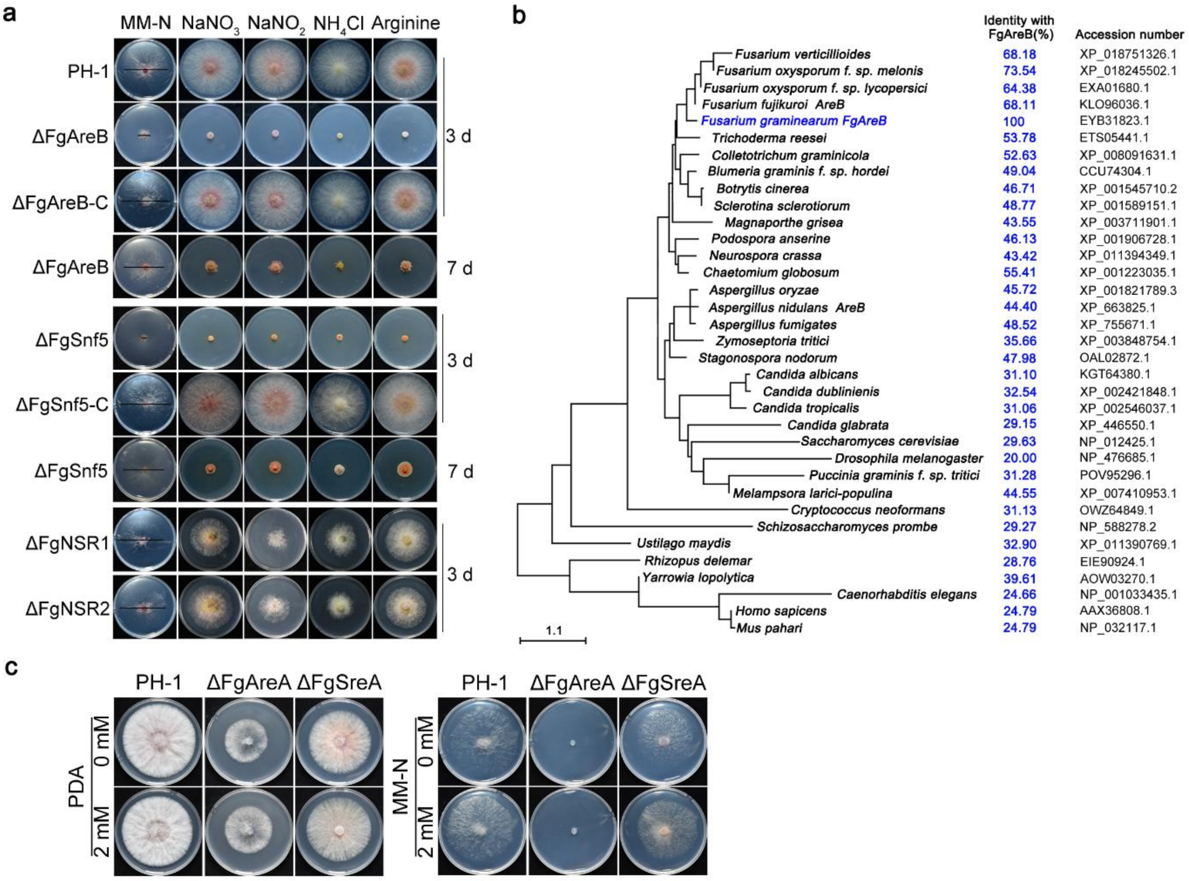


**Supplementary Fig. 2. Phylogenetic tree of FgAreB orthologs.**

**(a)** The ΔFgAreB, ΔFgSnf5, ΔFgNSR1, and ΔFgNSR2 mutants exhibited increased sensitivity to different nitrogen sources. A five-mm mycelial plug of PH-1 was inoculated on a MM-N plate supplemented with 10 mM $NaNO_3$, $NaNO_3$, arginine or

NH4Cl, and incubated at 25 °C for three and seven days.

**(b)** Phylogenetic tree was constructed based on deduced amino acid sequences of FgAreB and its orthologes with MEGA5 using the neighbor-joining method. The identity of FgAreB orthologs was analyzed using https://www.ebi.ac.uk/Tools/msa/clustalo/.

**(c)** The ΔFgAreA and ΔFgSreA mutants did not exhibit increased sensitivity to 2 mM SNP as compared with the wild-type PH-1. A five-mm mycelial plug of each strain was inoculated on a PDA or MM-N plate supplemented with 2 mM SNP at 25 °C for three days.

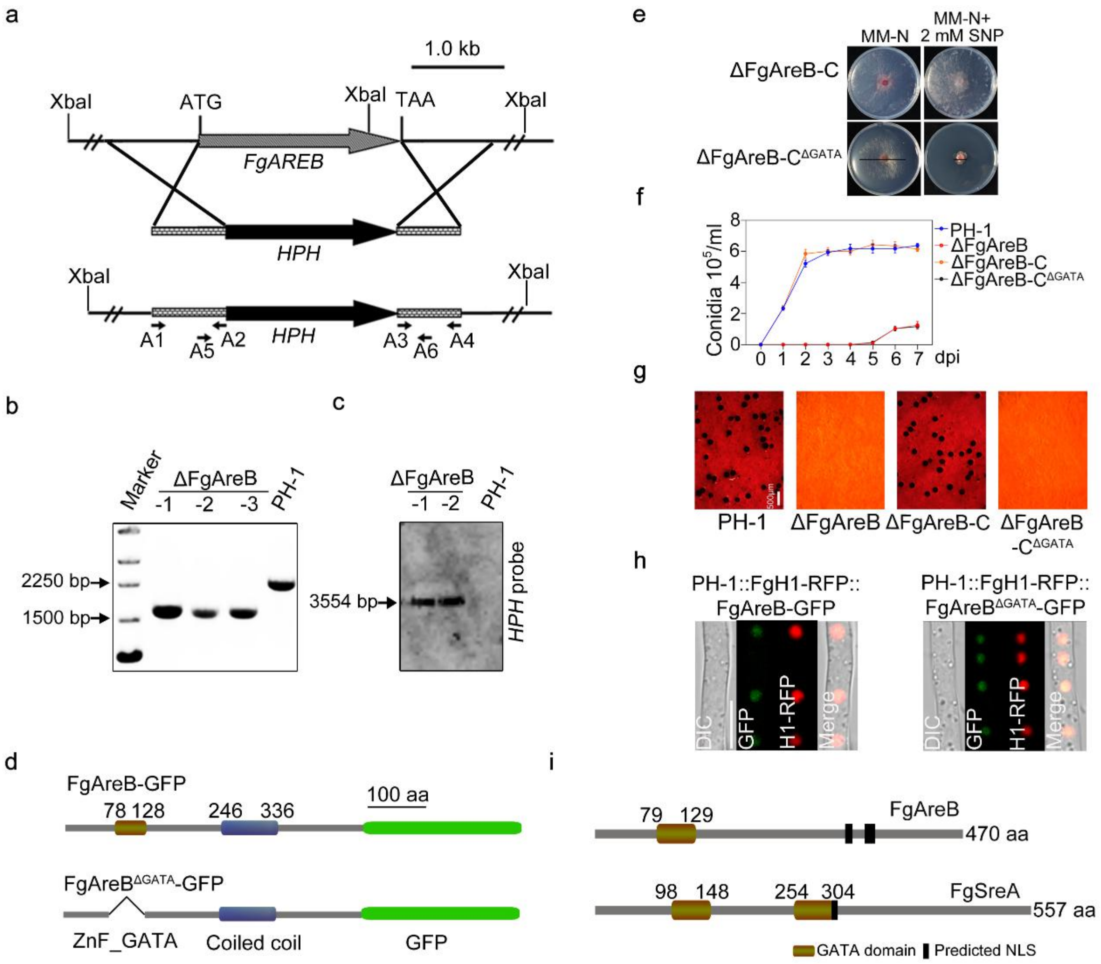


**Supplementary Fig. 3. Lack of the GATA binding domain of FgAreB inhibits *F. graminearum* growth and development.**

**(a)** Gene deletion strategy for FgAreB. Large black arrow indicates the hygromycin resistance cassette (*HPH*); arrows indicate primer binding sites (see Supplementary Data 10 for primer sequences).

**(b)** Confirmation of FgAreB deletion mutant (ΔFgAreB) by PCR. The FgAreB mutants were identified with the A5 + A6 primers (as shown in **(a)** schematic diagram and Supplementary Data 10) covering the replaced gene region in PH-1 and the *HPH* in the mutants. The experiment was repeated three times independently with similar results.

**(c)** Southern blot analysis of the FgAreB locus in PH-1 and ΔFgAreB. Genomic DNA was digested with Xba I. The experiment was repeated twice independently with similar results.

**(d)** Schematic map of FgAreB-GFP and FgAreB$^{\Delta GATA}$-GFP for constructing the ΔFgAreB-C (ΔFgAreB::FgAreB-GFP) and ΔFgAreB-C$^{\Delta GATA}$ (ΔFgAreB::FgAreB$^{\Delta GATA}$-GFP) complementation strains.

**(e)** The GATA domain of FgAreB is required for regulating sensitivity of *Fg* to SNP. A five-mm mycelial plug of each strain was inoculated on a solid MM-N plate supplemented with or without 2 mM SNP, and incubated at 25 °C for seven days. Black line indicates diameter of colony.

**(f)** ΔFgAreB-C$^{\Delta GATA}$ could not restore conidiation of ΔFgAreB. Conidia were quantified using a hemacytometer after incubation of the mutant in CMC for the days as indicated in the figure. Data are the mean ± standard errors from five biological replicates (n=5).

**(g)** The mutants ΔFgAreB and ΔFgAreB-C$^{\Delta GATA}$ exhibited sexual sterility. Each strain was grown on carrot agar for induction of perithecial formation. Bars: 500 μm. The experiment was repeated twice independently with similar results.

**(h)** ΔFgAreB-C and ΔFgAreB-C$^{\Delta GATA}$ localized to the nucleus. Bars: 10 μm. The experiment was repeated twice independently with similar results.

**(i)** Schematic map of GATA transcription factors FgAreB and FgSreA. The GATA domain with amino acid (aa) positions were labeled on the top and identified with http://smart.embl-heidelberg.de/smart/set_mode.cgi?NORMAL=1 database. The nucleus localization signals (NLS) were predicted with http://nls-mapper.iab.keio.ac.jp/cgi-bin/NLS_Mapper_form.cgi and https://rostlab.org/services/nlsdb/.

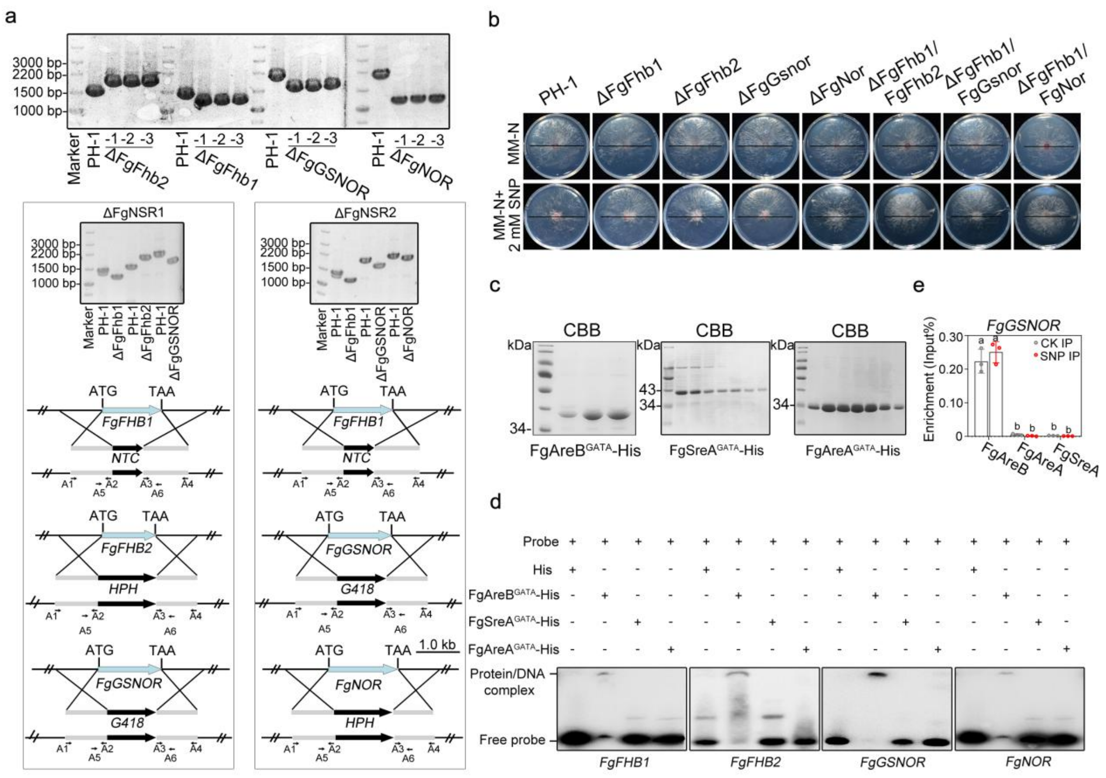

**Supplementary Fig. 4. Involvement of the *FgFHB1*, *FgFHB2*, *FgGSNOR* and *FgNOR* genes in NS response.**

**(a)** Strategy and confirmation for the *FgFHB1*, *FgFHB2*, *FgGSNOR, FgNOR* single deletion mutants, and triple mutants (ΔFgNSR1, ΔFgNSR2). The mutants were identified with A5+A6 primers (Supplementary Data 10) covering the replaced gene region in PH-1 and the corresponding antibiotic resistance cassette (*NTC*: Nourseothricin resistance cassette; *HPH*: hygromycin resistance cassette; *G418*: *Geneticin* resistance cassette) in the mutants. The experiment was repeated three times independently with similar results.

**(b)** Single and double deletion mutants of NS response genes exhibited slightly increased sensitivity to SNP. A five-mm mycelial plug of each strain was inoculated on a solid MM-N plate supplemented with or without 2 mM SNP and then incubated at 25 °C for three days. Black lines indicate diameter of colony.

**(c)** Purification of FgAreB$^{GATA}$-His, FgAreA$^{GATA}$-His and FgSreA$^{GATA}$-His and the proteins were stained by CBB. The experiment was repeated twice independently with similar results.

**(d)** Electrophoretic mobility shift assay (EMSA) showed that FgAreB, but not FgSreA and FgAreA, bound to the GATA *cis*-element (HGATAR) in the promoters of *FgFHB1*, *FgFHB2*, *FgGSNOR* and *FgNOR*. The experiment was repeated twice

independently with similar results.

**(e)** FgAreB, but not FgAreA and FgAreA, enriched at the *FgGSNOR* gene. The input- and ChIP-DNA samples from the strains treated with (SNP IP) or without (CK IP) 10 mM SNP were quantified by quantitative PCR assays with corresponding primer pairs (Supplementary Data 10). Secondary antibody rabbit IgG was used as a control. ChIP signals are shown as the percentages of input. Data presented are the mean ± standard errors from three biological replicates (n=3). Different letters represent statistically significant differences according to the one-way ANOVA test ($p < 0.05$).

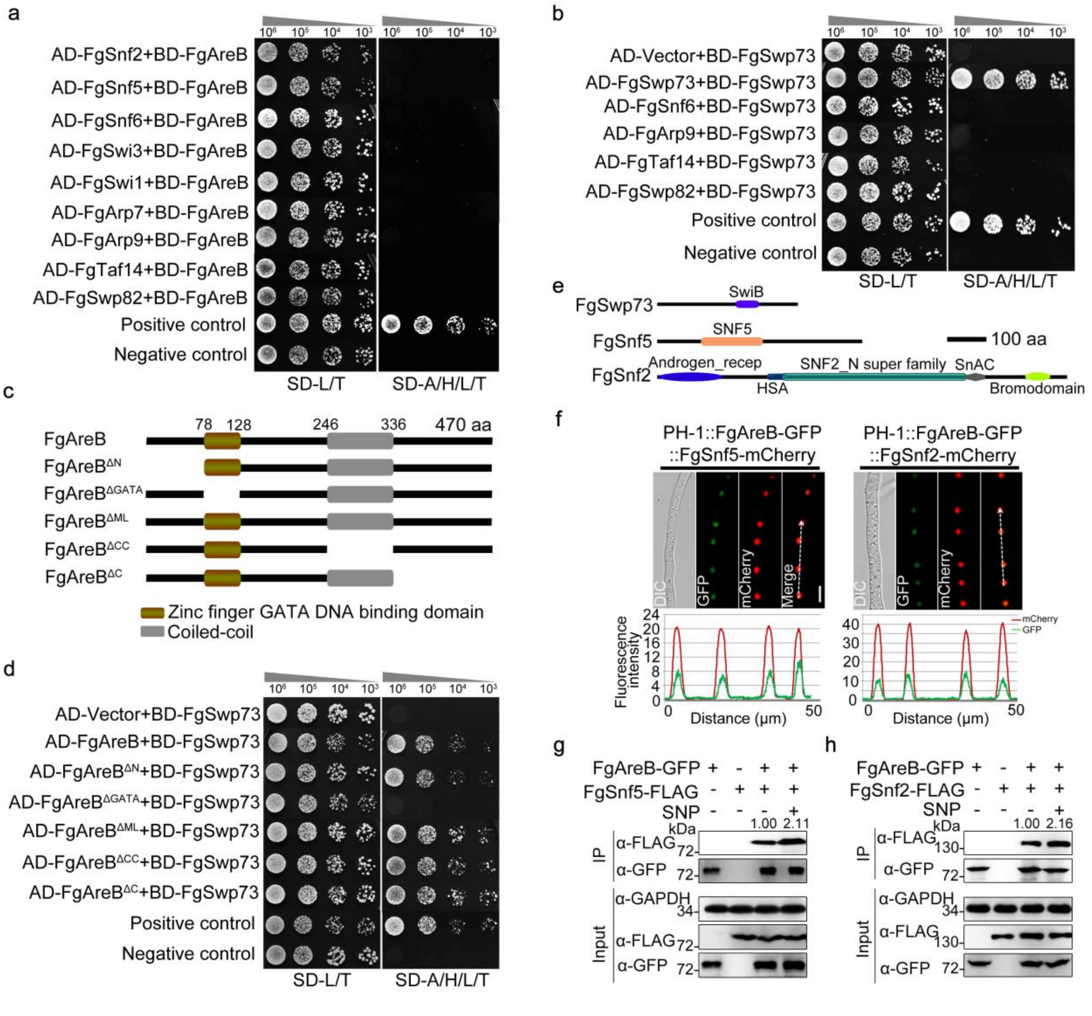


**Supplementary Fig. 5. FgAreB associates with the SWI/SNF complex both *in vivo* and *in vitro*.**

**(a)** FgAreB interacts with FgSwp73 (Fig. 3a), but not with other subunits of SWI/SNF complex in Y2H assay. Serial dilutions of the yeast cells were plated on SD-L/T/H/A. The experiment was repeated three times independently with similar results.

**(b)** FgSwp73 did not interact with four subunits of the SWI/SNF complex in Y2H

assay. The experiment was repeated three times independently with similar results.

**(c)** Schematic map of FgAreB and truncated FgAreB. Yellow and grey represent the conserved GATA zinc finger domain and coiled-coil domain of FgAreB, respectively.

**(d)** The GATA domain of FgAreB is required for its interaction with FgSwp73 in Y2H assay. The interactions between different truncated FgAreB and FgSwp73 were determined by Y2H assay. The experiment was repeated three times independently with similar results.

**(e)** Schematic structures of the SWI/SNF complex subunits FgSwp73, FgSnf5 and FgSnf2.

**(f)** FgAreB-GFP co-localized with FgSnf5-mCherry and FgSnf2-mCherry into the nucleus. The wild-type background PH-1 strain containing FgAreB-GFP and FgSnf5-mCherry or FgSnf2-mCherry was examined using a confocal microscopy (upper panel). Bars: 10 μm. Co-localization of the proteins was evaluated by line scan graph analysis (lower panel). White dotted arrow indicates the analyzed area. The experiment was repeated twice independently with similar results.

**(g)** FgAreB was associated with FgSnf5 *in vivo* by Co-IP assays. Proteins were extracted from the corresponding strains growth in YEPD for 16 hr and then cultured for 4 hr in MM-N with or without 10 mM SNP. The protein samples were immunoprecipitated with anti-GFP agarose beads, and subsequently immunoblotted with anti-FLAG or anti-GFP antibody (upper panel). Lower panels show the Input control. The experiment was repeated twice independently with similar results.

**(h)** FgAreB was associated with FgSnf2 *in vivo* by Co-IP assays. The assays were performed as in **(g)**.

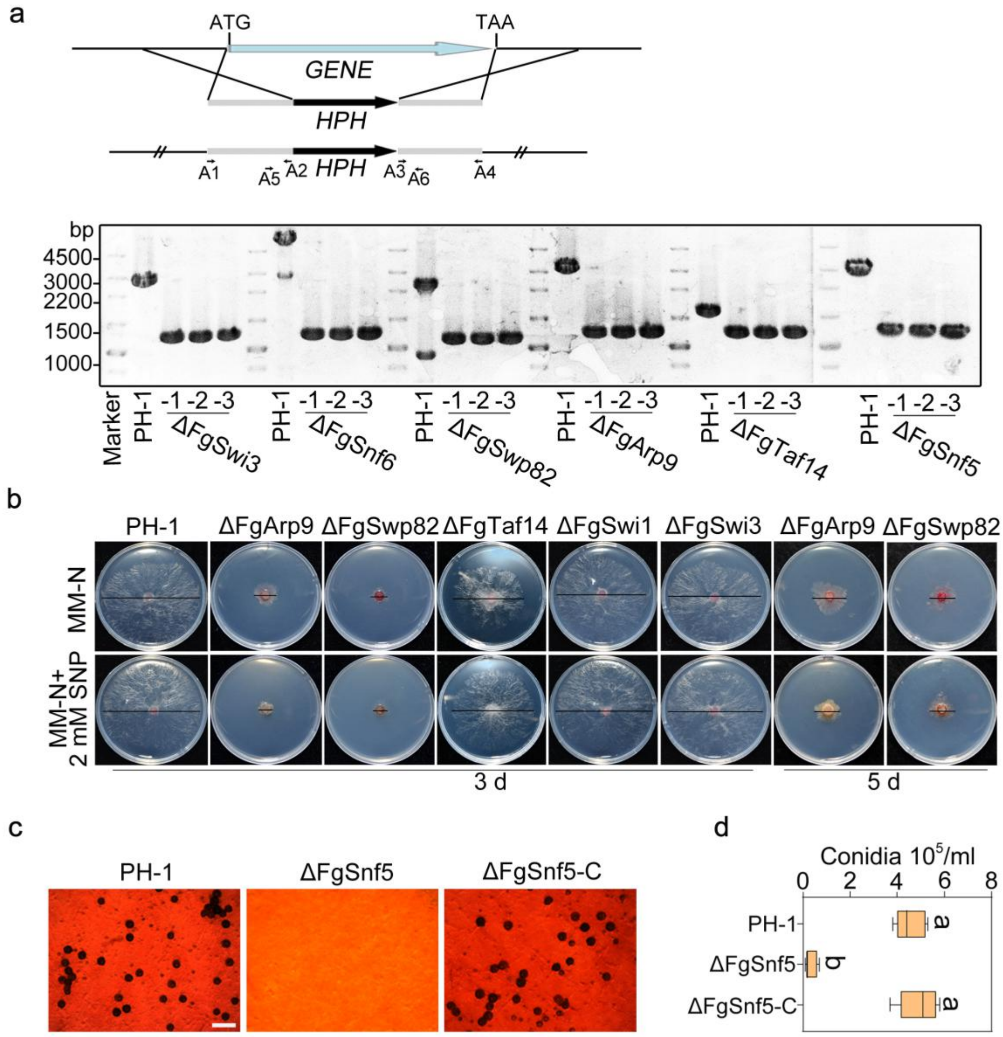


**Supplementary Fig. 6. The core subunit of SWI/SNF complex is involved in nitrosative stress and fungal growth.**

**(a)** Confirmation of FgSwi3, FgSnf6, FgSwp82, FgArp9, FgTaf14, and FgSnf5 deletion mutants by PCR. The up-panel showed gene deletion strategy for SWI/SNF complex subunits and the deletion mutants were identified with A5+A6 primers (Supplementary Data 10) that cover the gene replacement region in WT and selection marker genes in mutant (*HPH*: hygromycin resistance gene). The experiment was repeated three times independently with similar results.

**(b)** Sensitivity of the SWI/SNF complex deletion mutants to SNP. A five-mm mycelial plug of each strain was inoculated on a solid MM-N supplement with 0 or 2 mM SNP and then incubated at 25 °C for three and five days. Black lines indicate diameter of colony.

**(c)** Deletion of FgSnf5 led to sexual sterility. Each strain was grown on carrot agar for induction of perithecial formation. Bars: 500 μm. The experiment was repeated twice

independently with similar results.

**(d)** Deletion of FgSnf5 caused reduced conidiation. Conidia were quantified using a hemacytometer after incubation in CMC for four days. Data are shown as box plots with the interquartile range as the upper and lower confines of the box, and the median as a solid line within the box. Different letters indicate statistically significant differences according to the one-way ANOVA test ($p$ <0.05).

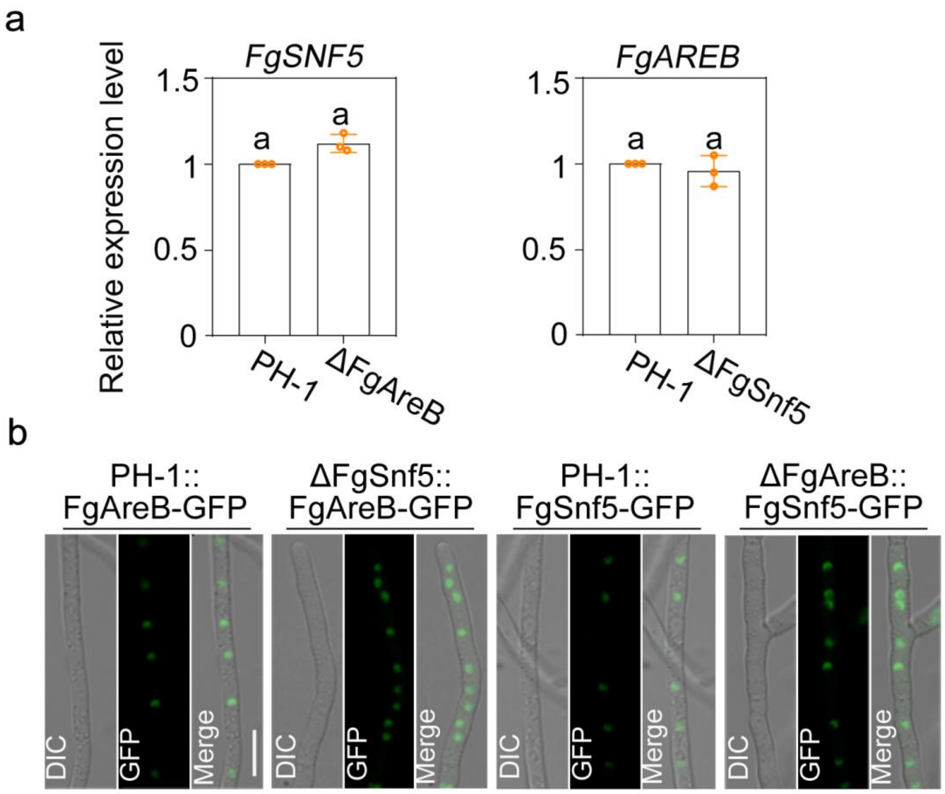


**Supplementary Fig. 7. Deletion of *FgAreB* or *FgSnf5* does not affect each other's expression.**

**(a)** No expression changes were observed for *FgSNF5* or *FgAREB* in the corresponding gene deletion strain as compared to that in the wild-type PH-1. Data presented are the mean ± standard errors from three biological replicates (n=3). The same letter indicate no statistically significant differences according to two-tailed Student's $t$-test ($p$ ≥0.05).

**(b)** Deletion of *FgAreB* or *FgSnf5* did not affect each other's subcellular localization. Confocal images of hyphae showing nuclear localization of FgAreB-GFP and FgSnf5-GFP in the strains tested. Bars: 10 μm. The experiment was repeated twice independently with similar results.

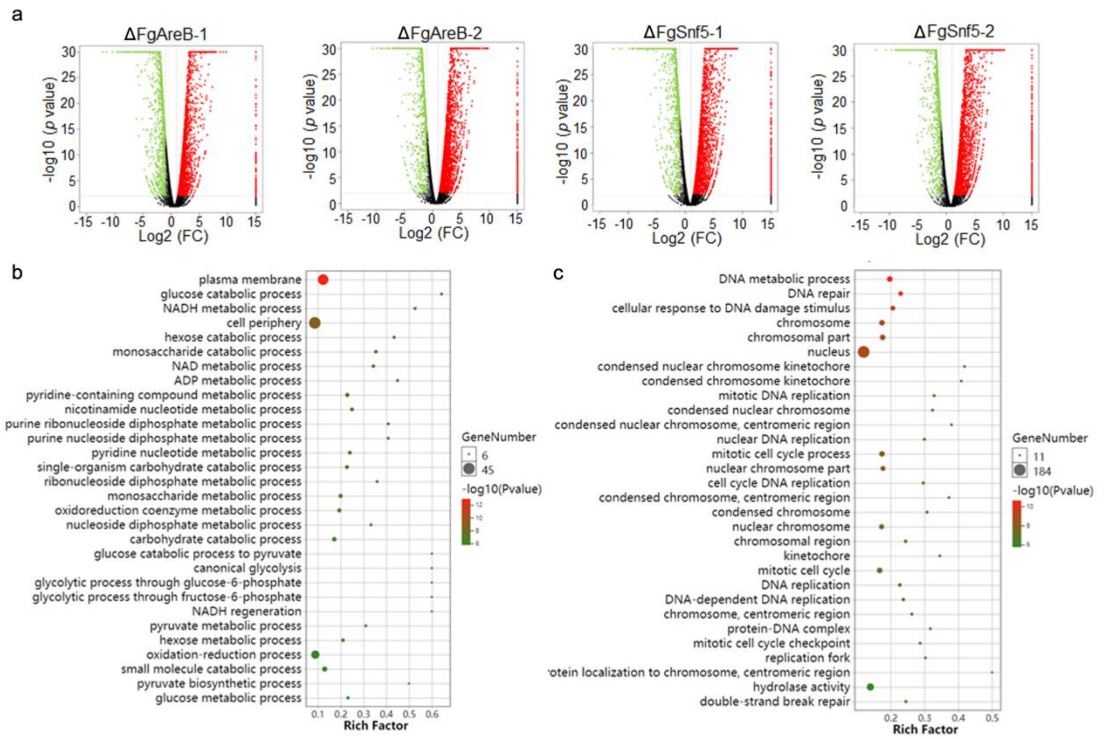


**Supplementary Fig. 8. FgSnf5 and FgAreB co-regulate some sets of genes.**

**(a)** Volcano plots indicate differentially expressed genes between the mutants and PH-1. Red and green dots indicate up- and down-regulated genes, respectively.

**(b)** Gene ontology enrichment analysis of down-regulated genes in both ΔFgAreB and ΔFgSnf5. Significantly enriched GO categories are listed in Supplementary Data 8.

**(c)** Gene ontology enrichment analysis of up-regulated genes in both ΔFgAreB and ΔFgSnf5. Significant enriched GO categories are listed in Supplementary Data 9.

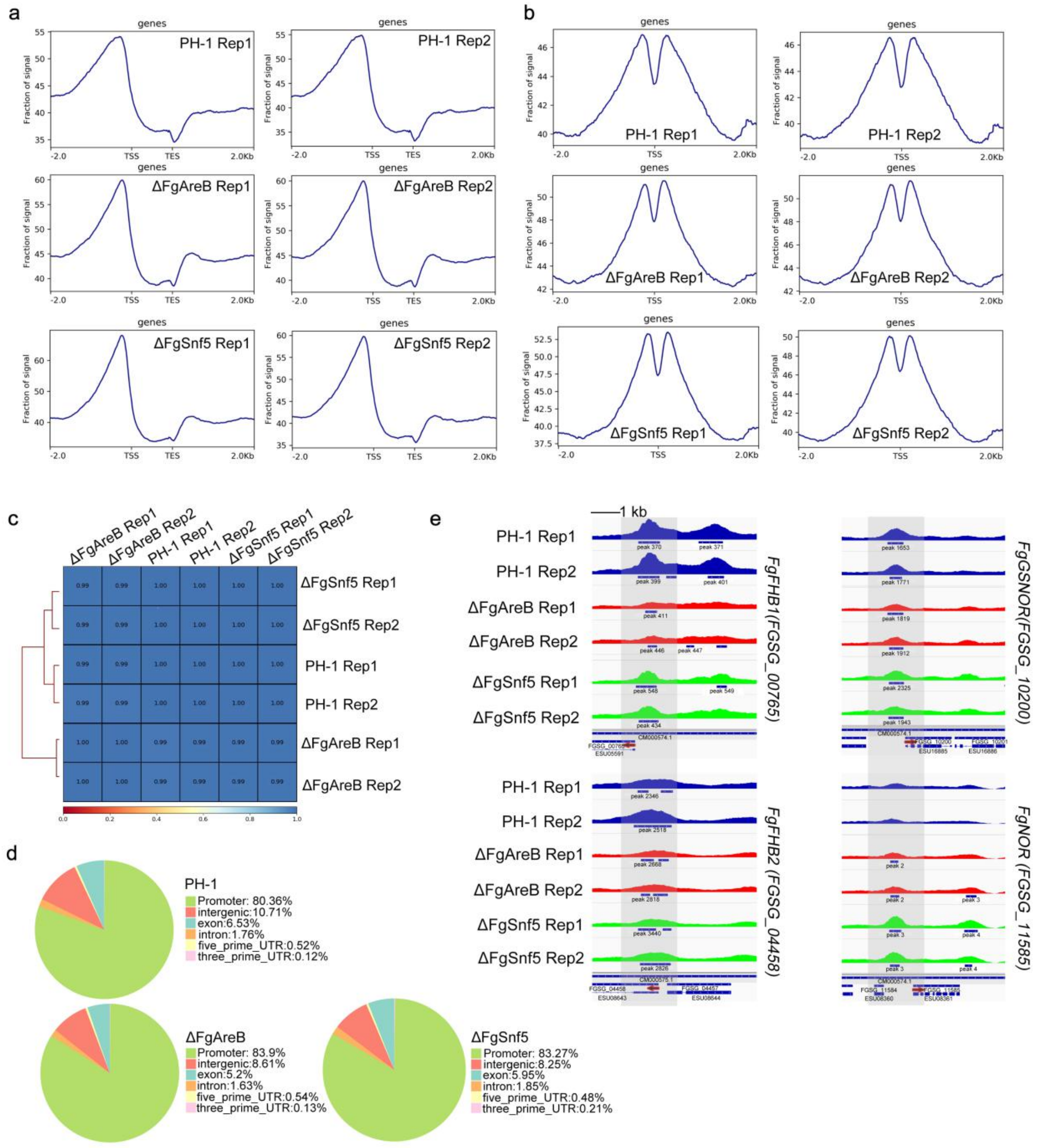


**Supplementary Fig. 9. Chromatin landscape profiles of ΔFgAreB and ΔFgSnf5.**

**(a)** Distribution of the ATAC-seq reads in 2 kb up- and down-stream of TES in PH-1, ΔFgAreB and ΔFgSnf5**.** TES, Transcription End Site.

**(b)** Distribution of the ATAC-seq reads in 2 kb up- and down-stream of TSS in PH-1, ΔFgAreB and ΔFgSnf5. TSS, Transcription Start Site.

**(c)** Correlation between ATAC-seq replicates for PH-1, ΔFgAreB and ΔFgSnf5 were calculated by the deeptools according to raw bam files in 10-kb genomic bins**.**

**(d)** Genomic distribution of accessible peaks in PH-1, ΔFgAreB, and ΔFgSnf5**.**

**(e)** Genomic landscape of NS response genes in PH-1, ΔFgAreB, and ΔFgSnf5.

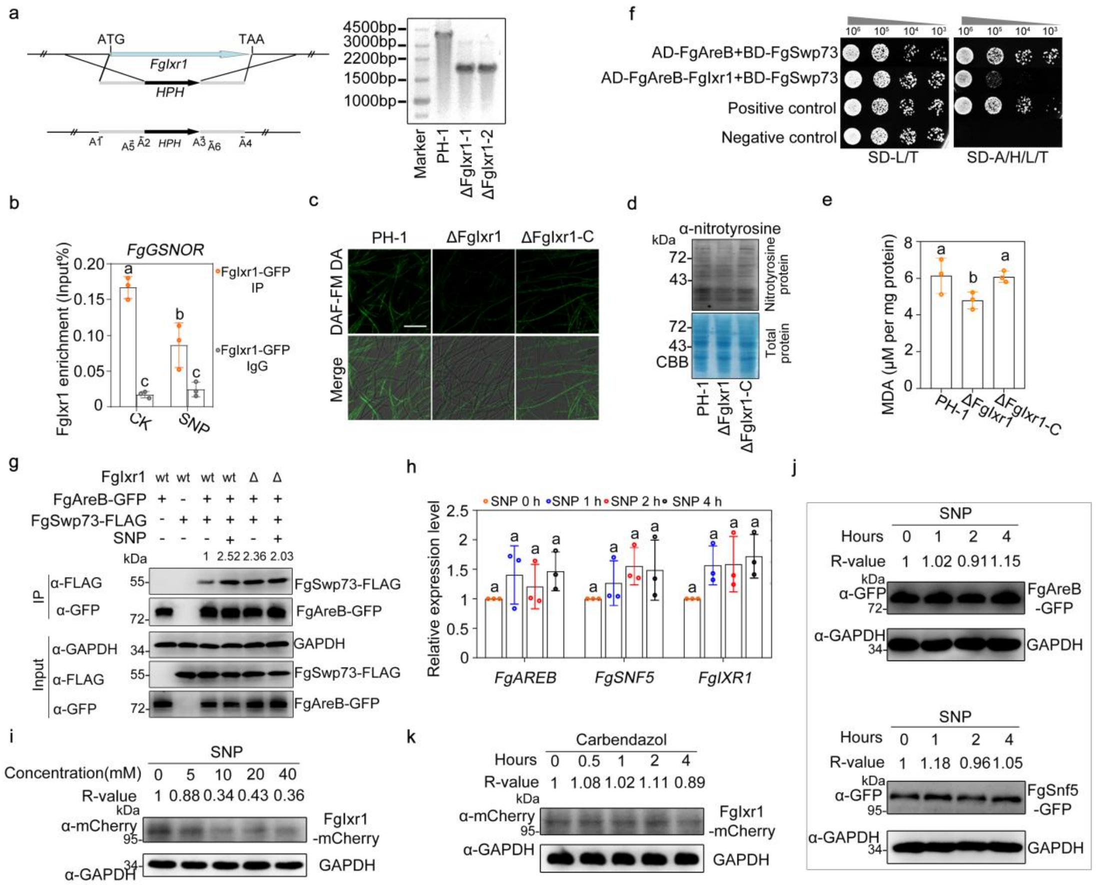


**Supplementary Fig. 10. FgIxr1 is involved in regulating NS response in *F. graminearum*.**

**(a)** Confirmation of FgIxr1 deletion mutants by PCR. The up-panel showed gene deletion strategy for FgIxr1 and the mutants was identified with A5+A6 primers (Supplementary Data 10) by PCR (lower panel) that cover replaced region of gene in wild type PH-1 and the selection marker genes in mutants. The experiment was repeated three times independently with similar results.

**(b)** Enrichment of FgIxr1 decreased at the promoter of the *FgGSNOR* gene under SNP treatment. DNA sample was extracted from ΔFgIxr1::FgIxr1-GFP that was cultured in YEPD for 16 hr and then incubated for 4 hr in MM-N without or with 10 mM SNP. ChIP- and input-DNA samples were quantified by quantitative PCR assays with corresponding primer pairs (Supplementary Data 10). Secondary rabbit IgG used as a control. ChIP signals are shown as the percentages of the input. Data presented are the mean ± standard errors from three biological replicates (n=3). Different letters indicate statistically significant differences according to the one-way ANOVA test ($p$ <0.05).

**(c)** ΔFgIxr1 produced less NO compared with PH-1 under SNP treatment. Mycelia

that were cultured in YEPD for 16 hr and then incubated for 4 hr in MM-N with 10 mM SNP were stained with DAF-FM DA. Fluorescence was detected by confocal microscopy. Bars: 50 μm. The experiment was repeated three times independently with similar results.

**(d)** ΔFgIxr1 exhibited decreased nitrotyrosine level under SNP treatment. Protein sample was extracted from each strain that was cultured in YEPD for 16 hr and then incubated for 4 hr in MM-N with 10 mM SNP. Nitrotyrosine was detected by western blot with anti-nitrotyrosine polyclonal antibody. Lower panel: Protein loading control stained by CBB. The experiment was repeated three times independently with similar results.

**(e)** ΔFgIxr1 showed decreased level of malondialdehyde (MDA) under SNP treatment. Data presented are the mean ± standard errors from three biological replicates (n=3). Different letters represent statistically significant differences according to the one-way ANOVA test ($p$ <0.05).

**(f)** FgIxr1 interfered with FgAreB-FgSwp73 interaction in Y2H assay. Serial dilutions of yeast cells expressing the constructs as indicated were grown on SD-L/T/H/A. The experiment was repeated three times independently with similar results.

**(g)** Deletion of FgIxr1 enhanced the FgAreB-FgSwp73 association. Proteins were extracted from the corresponding strain that were cultured in YEPD for 16 hr and then incubated for 4 hr in MM-N with or without 10 mM SNP. Protein samples were immunoprecipitated with anti-GFP agarose beads, and subsequently immunoblotted with anti-FLAG or anti-GFP antibody (upper panel). Lower panels show the input control. "wt" and "Δ" indicate the wild type and FgIxr1 deletion mutant, respectively, containing (+) or not containing (-) the corresponding protein. The experiment was repeated twice independently with similar results.

**(h)** SNP treatment does not affect the transcription of *FgAREB*, *FgSNF5*, and *FgIXR1*. PH-1 was cultured in YEPD for 16 hr and then transferred to MM-N with 10 mM SNP for the time points as indicated. The *FgACTIN* gene used as internal control. Data presented are the mean ± standard errors from three biological replicates (n=3). Same letters represent no statistically significant differences according to the one-way ANOVA test ($p \geq 0.05$).

**(i)** FgIxr1-mCherry remained a certain level after treatment with SNP at 10 mM. Protein samples were extracted from ΔFgIxr1::FgIxr1-mCherry that was cultured in

YEPD for 16 hr and then incubated for 4 hr in MM-N containing 5, 10, 20 or 40 mM SNP. The amount of FgIxr1-mCherry protein was detected by anti-mCherry antibody. GAPDH served as a loading control. The intensity of the FgIxr1-mCherry band at each time-point is relative to the amount of FgIxr1-mCherry before SNP treatment (0 mM). The experiment was repeated twice independently with similar results.

**(j)** Protein levels of FgSnf5-GFP and FgAreB-GFP were not affected by SNP treatment. ΔFgSnf5::FgSnf5-GFP and ΔFgAreB::FgAreB-GFP strains were cultured in YEPD for 16 hr and then incubated in MM-N supplemented with 10 mM SNP for time points as indicated in the figure. Protein level of FgAreB-GFP (upper panel) and FgSnf5-GFP (lower panel) were detected by western blot with anti-GFP antibody. GAPDH served as a loading control. The intensity of the FgSnf5-GFP or FgAreB-GFP band at each time-point is relative to the amount of FgSnf5-GFP or FgAreB-GFP before SNP treatment (0 hr), respectively. The experiment was repeated twice independently with similar results.

**(k)** Fungicide carbendazim does not promote degradation of FgIxr1-mCherry. Protein samples were extracted from ΔFgIxr1::FgIxr1-mCherry that was cultured in YEPD for 16 hr and then transferred to MM-N containing 1 μg/ml carbendazol for time points as indicated. Protein level of FgIxr1-mCherry was detected by western blot with anti-mCherry antibody. GAPDH served as a loading control. The intensity of the FgIxr1-mCherry band at each time-point is relative to the amount of FgIxr1-mCherry before carbendazol treatment (0 hr). The experiment was repeated twice independently with similar results.

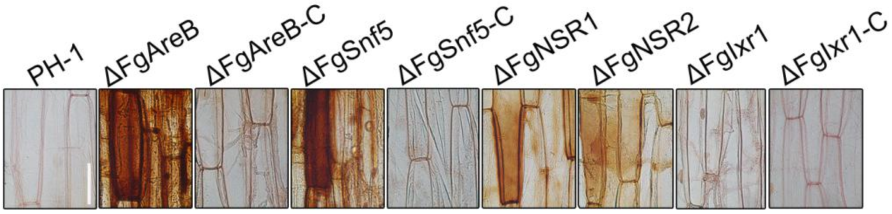


**Supplementary Fig. 11. ΔFgAreB, ΔFgSnf5, ΔFgNSR1, ΔFgNSR2 show increased ROS accumulation.**

The mutants ΔFgAreB, ΔFgSnf5, ΔFgNSR1 and ΔFgNSR2, but not ΔFglxr1 provoked more ROS production in the wheat cell during infection as compred to the wild-type PH. ROS accumulation in cells of coleoptile was assayed by 3, 3-diaminobenzidine (DAB) staining at 2 dpi inoculated with each strain. Bars: 500

μm. The experiment was repeated twice independently with similar results.

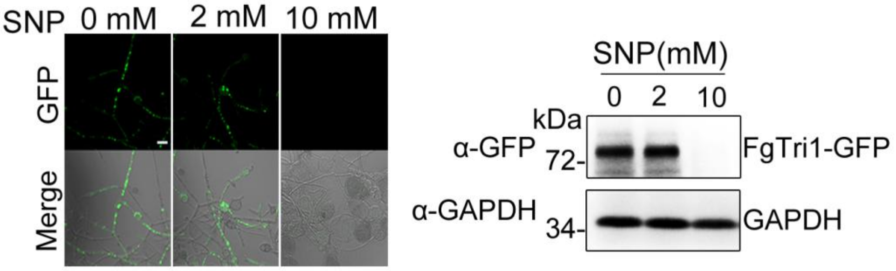


**Supplementary Fig. 12. Toxisome formation is inhibited by SNP treatment**.

Toxisome formation was examined in the strain ΔFgTri1::FgTri1-GFP that was cultured in the toxin inducing medium (TBI) containing 0, 2 or 10 mM SNP (left panel). Bars: 20 μm. FgTri1-GFP protein level was determined by western blot with anti-GFP antibody (right panel). GAPDH used as loading control. The experiment was repeated twice independently with similar results.

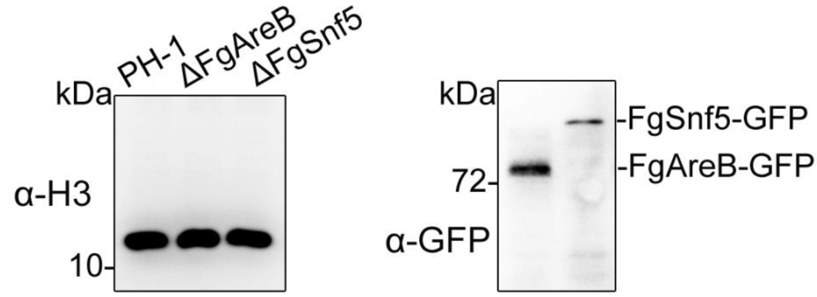


**Supplementary Fig. 13. ChIP grade antibodies used for ChIP-qPCR assays were validated by western blotting.**

Protein samples extracted from PH-1, ΔFgAreB and ΔFgSnf5 strains as well as ΔFgAreB::FgAreB-GFP or ΔFgSnf5::FgSnf5-GFP were detected by western blotting with ChIP grade antibodies anti-H3 and anti-GFP, respectively.

**Supplementary Table 1.** Subunits of SWI/SNF complex in *F. graminearum*

| **SWI/SNF in yeast** | **Homologs in *F.graminearum*** | **Function** |
|---|---|---|
| Swi2/Snf2 | FGSG_07306 | Contains DNA-stimulated ATPase activity; functions interdependently in transcriptional activation with Snf5p and Snf6p |
| Swp73/SNF12 | FGSG_10724 | Involved in transcriptional regulation |
| Swi1 | FGSG_08487 | Regulates transcription by remodeling chromatin; self-assembles to form Swi prion and to alter expression pattern |
| Snf5 | FGSG_06954 | Involved in transcriptional regulation; functions interdependently in transcriptional activation with Snf2p and Snf6p |
| Swi3 | FGSG_01925 | Contains SANT domain that is required for SWI/SNF assembly; is essential for displacement of histone H2A-H2B dimers during ATP-dependent remodeling |
| Swp82 | FGSG_01193 | Has an as yet unidentified role in the complex |
| Swp61/Arp7 | FGSG_00874 | Actin-related protein involved in transcriptional regulation |
| Swp59/Arp9 | FGSG_06172 | Actin-related protein involved in transcriptional regulation |
| Snf6 | FGSG_00856 | Functions interdependently in transcriptional activation with Snf2p and Snf5p |
| Swp29/Taf14 | FGSG_04108 | Involved in RNA polymerase II transcription initiation and in chromatin modification; contains a YEATS domain |
| Snf11 | without homolog | Involved in transcriptional regulation; interacts with a highly conserved 40-residue sequence of Snf2p |
| Rtt102 | without homolog | Suggested role in chromosome maintenance; possible weak regulator of Ty1 transposition |

**Supplementary Table 2.** Fold change of FgNSRs in ΔFgAreB and ΔFgSnf5 mutants by RNA-seq in *F. graminearum.*

| **Accession number** | **Gene name** | **ΔFgAreB logFC** | | | | **ΔFgSnf5 logFC** | | | |
|---|---|---|---|---|---|---|---|---|---|
| | | Repeat 1 | *p*-value | Repeat 2 | *p*-value | Repeat 1 | *p*-value | Repeat 2 | *p*-value |
| FGSG_00765 | *FgFHB1* | -2.83 | 1.02e-46 | – | – | -6.1 | 9.44e-102 | -5.6 | 1.51e-89 |
| FGSG_04458 | *FgFHB2* | -4.54 | 8.17e-94 | -3.8 | 4.00e-77 | -5.1 | 2.14e-109 | -6.14 | 6.46e-128 |
| FGSG_10200 | *FgGSNOR* | – | – | – | – | – | – | – | – |
| FGSG_11585 | *FgNOR* | -2.59 | 1.59e-36 | -5.2 | 8.85e-74 | -4.5 | 3.55e-64 | -4.5 | 3.10e-58 |
| FGSG_08977 | *FgHEMC* | – | – | – | – | – | – | – | – |
