# Supplementary material for: Interplay of two transcription factors for recruitment of the chromatin remodeling complex modulates fungal nitrosative stress response
Source: Nat Commun. 2021 May 6;12:2576. doi: 10.1038/s41467-021-22831-8 (PMC8102577; doi:10.1038/s41467-021-22831-8)
Supplement: Supplementary file 3 — Description of Additional Supplementary Files [file 41467_2021_22831_MOESM3_ESM.pdf]

Description of Additional Supplementary Files

File Name: Supplementary Data 1
Description: The gene IDs of partial *Fg* mutant library.

File Name: Supplementary Data 2
Description: A list of putative FgAreB-interacting proteins identified by yeast two hybrid screens.

File Name: Supplementary Data 3
Description: The down-regulated genes in FgAreB mutant.

File Name: Supplementary Data 4
Description: The down-regulated genes in FgSnf5 mutant.

File Name: Supplementary Data 5
Description: The down-regulated genes in both FgSnf5 and FgAreB mutants.

File Name: Supplementary Data 6
Description: The up-regulated genes in FgAreB mutant.

File Name: Supplementary Data 7
Description: The up-regulated genes in FgSnf5 mutant.

File Name: Supplementary Data 8
Description: The up-regulated genes in both FgSnf5 and FgAreB mutants.

File Name: Supplementary Data 9
Description: GO enrichment analysis of down-regulated genes in both FgAreB and FgSnf5 mutants.

File Name: Supplementary Data 10
Description: GO enrichment analysis of up-regulated genes in both FgAreB and FgSnf5 mutants

File Name: Supplementary Data 11
Description: A list of PCR primers used in this study and their relevant characteristics.
