# Supplementary material for: Interplay of two transcription factors for recruitment of the chromatin remodeling complex modulates fungal nitrosative stress response
Source: Nat Commun. 2021 May 6;12:2576. doi: 10.1038/s41467-021-22831-8 (PMC8102577; doi:10.1038/s41467-021-22831-8)
Supplement: Supplementary file 15 — Reporting Summary [file 41467_2021_22831_MOESM15_ESM.pdf]

nature research

Corresponding author(s): Zhonghua Ma

Last updated by author(s): Mar 10, 2021

Reporting Summary

Nature Research wishes to improve the reproducibility of the work that we publish. This form provides structure for consistency and transparency in reporting. For further information on Nature Research policies, see our Editorial Policies and the Editorial Policy Checklist.

Statistics

For all statistical analyses, confirm that the following items are present in the figure legend, table legend, main text, or Methods section.

n/a	Confirmed	
☐	☒	The exact sample size (n) for each experimental group/condition, given as a discrete number and unit of measurement
☐	☒	A statement on whether measurements were taken from distinct samples or whether the same sample was measured repeatedly
☐	☒	The statistical test(s) used AND whether they are one- or two-sided<br>*Only common tests should be described solely by name; describe more complex techniques in the Methods section.*
☒	☐	A description of all covariates tested
☒	☐	A description of any assumptions or corrections, such as tests of normality and adjustment for multiple comparisons
☐	☒	A full description of the statistical parameters including central tendency (e.g. means) or other basic estimates (e.g. regression coefficient) AND variation (e.g. standard deviation) or associated estimates of uncertainty (e.g. confidence intervals)
☐	☒	For null hypothesis testing, the test statistic (e.g. F, t, r) with confidence intervals, effect sizes, degrees of freedom and P value noted<br>*Give P values as exact values whenever suitable.*
☒	☐	For Bayesian analysis, information on the choice of priors and Markov chain Monte Carlo settings
☒	☐	For hierarchical and complex designs, identification of the appropriate level for tests and full reporting of outcomes
☒	☐	Estimates of effect sizes (e.g. Cohen's d, Pearson's r), indicating how they were calculated

Our web collection on statistics for biologists contains articles on many of the points above.

Software and code

Policy information about availability of computer code

Data collection	The RNA-seq data were collected by Illumina NovaSeq 6000. The ATAC-seq data were collected by Illumina HiSeq X Ten.
Data analysis	Clean reads were mapped to the F. graminearum genome by Hisat2 (version 2.1.0). Samtools (version 1.3.1) was used to remove potential PCR duplicates, and MACS2 software (version 2.1.1.20160309) was used to call peaks by default parameters (nomodel; shift -100; extsize 200; model fold, 5, 50; qvalue, 0.05). Adapters and low-quality reads were filtered out through Trimmomatic (version 0.38). The diffpeak was identified using Diffbind (version 1.16.3). Heatmap of regulatory region distribution was obtained using Deeptool (version 3.2.1).

For manuscripts utilizing custom algorithms or software that are central to the research but not yet described in published literature, software must be made available to editors and reviewers. We strongly encourage code deposition in a community repository (e.g. GitHub). See the Nature Research guidelines for submitting code & software for further information.

Data

Policy information about availability of data

All manuscripts must include a data availability statement. This statement should provide the following information, where applicable:

- Accession codes, unique identifiers, or web links for publicly available datasets
- A list of figures that have associated raw data
- A description of any restrictions on data availability

Relevant data supporting the findings of this study are available in this article and its Supplementary Information files. The ATAC-Seq and RNA-Seq data have been deposited in the NCBI BioProject database with accession code PRJNA648257. Source data are provided with this paper. GATA zinc finger domain and coiled-coil domain were predicted according to the SMART protein database (http://smart.embl-heidelberg.de) and NBCI protein database (https://blast.ncbi.nlm.nih.gov/Blast.cgi). The identity of FgAreB orthologs was analyzed using https://www.ebi.ac.uk/Tools/msa/clustalo/. The GATA domain with amino acid (aa) positions were labeled on the top and identified with http://smart.embl-heidelberg.de/smart/set_mode.cgi?NORMAL=1 database. The nucleus localization signals (NLS) were

predicted with http://nls-mapper.iab.keio.ac.jp/cgi-bin/NLS_Mapper_form.cgi and https://rostlab.org/services/nlsdb/.

Field-specific reporting

Please select the one below that is the best fit for your research. If you are not sure, read the appropriate sections before making your selection.

- [x] Life sciences
- [ ] Behavioural & social sciences
- [ ] Ecological, evolutionary & environmental sciences

For a reference copy of the document with all sections, see nature.com/documents/nr-reporting-summary-flat.pdf

Life sciences study design

All studies must disclose on these points even when the disclosure is negative.

Sample size	Sample sizes are noted in the Figure legends. No statistical methods were used to predetermine sample size. Comparable sample sizes were chosen that allow data reproducibility for each experimental conditions. For all data, positive and negative controls were included whenever possible. The sample sizes provided for each experiment are typical and determined based on previous published researches.
Data exclusions	No data were excluded
Replication	All attempts at replication were successful. The number of replicates is indicated in the corresponding figure legend and/or in the Method section.
Randomization	Randomization was used for all the biological experiments.
Blinding	No blinding was done as none of the experiments described in this study involves group allocation during data collection or analyses.

Reporting for specific materials, systems and methods

We require information from authors about some types of materials, experimental systems and methods used in many studies. Here, indicate whether each material, system or method listed is relevant to your study. If you are not sure if a list item applies to your research, read the appropriate section before selecting a response.

Materials & experimental systems

n/a	Involved in the study	
[ ]	[x]	Antibodies
[x]	[ ]	Eukaryotic cell lines
[x]	[ ]	Palaeontology and archaeology
[x]	[ ]	Animals and other organisms
[x]	[ ]	Human research participants
[x]	[ ]	Clinical data
[x]	[ ]	Dual use research of concern

Methods

n/a	Involved in the study	
[x]	[ ]	ChIP-seq
[x]	[ ]	Flow cytometry
[x]	[ ]	MRI-based neuroimaging

Antibodies

Antibodies used	Rabit polyclonal anti-GFP (ab290, Abcam, Cambridge, UK; 1:500 dilution) antibody; Rabit monoclonal anti-H3 (ab1791, Abcam, Cambridge, UK; 1:500 dilution) for ChIP-qPCR; Mouse monoclonal anti-FLAG (A9044, Sigma, St. Louis, MO, USA, 1:5000 dilution); Rabit polyclonal anti-GFP (ab32146, Abcam, Cambridge, UK, 1:5000 dilution); Mouse monoclonal anti-mCherry (ab125096, Abcam, Cambridge, UK, 1:2000 dilution); Mouse monoclonal anti-GAPDH antibody (EM1101, Hua An Biotech. Ltd., Hangzhou, China, 1:5000 dilution); Mouse monoclonal anti-GST (M0807, Hangzhou Hua An Biotechnology Co., Ltd., 1:1000 dilution); Mouse monoclonal anti-His (ab18184, Abcam, Cambridge, MA, USA, 1:1000 dilution); Rabbit polyclonal anti-nitrotyrosine antibody at 1:1000 dilution (A-21285, Thermo Scientific); secondary antibody Goat polyclonal anti-rabbit IgG-HRP (HA1001, Hua An Biotech. Ltd., Hangzhou, China, 1:5000 dilution); Goat polyclonal anti-mouse IgG-HRP (HA1006, Hua An Biotech. Ltd., Hangzhou, China, 1:5000 dilution).
Validation	All primary antibodies used are commercial tag antibodies or anti conserved protein antibodies (For example, H3 and GAPDH). The validation of GFP and H3 antibodies used in ChIP-qPCR assays are presented in Supplementary Figure13. Other primary tag antibodies (such as, FLAG, GFP, mCherry, GST, HIS) used in this study were commercially validated on the manufacturer's websites and were also validated in our previous studies (Yin et al, New Phytologist, 2018; Yin et al, Plos Pathogens, 2018; Liu et al, Nature Communications, 2019). The anti-nitrotyrosine was referred to a previous work in Magnaporthe oryzae (Marroquin-Guzman et al, Nature Microbiology, 2017).
